# Surface Display of Type 1 Fimbriae on *Shigella flexneri* Induces Antigen-Specific Immune Response via Oral Route

**DOI:** 10.3390/vaccines13030280

**Published:** 2025-03-06

**Authors:** Shuli Sang, Rui Yu, Yunyun Mao, Yanfang Zhai, Chen Cao, Kai Li, Yiyan Guan, Haoxia Tao, Chunjie Liu, Yanchun Wang

**Affiliations:** Laboratory of Advanced Biotechnology, Beijing Institute of Biotechnology, 20 Dongda Street, Fengtai District, Beijing 100071, China; sangshuli@bmi.ac.cn (S.S.); yurui1102@139.com (R.Y.); myy-0706@163.com (Y.M.); 17888838150@163.com (Y.Z.); 1021226001@tju.edu.cn (C.C.); 13910096703@139.com (K.L.); yiyiyiyan610@163.com (Y.G.); taohaoxia@126.com (H.T.)

**Keywords:** type 1 fimbriae, attenuated bacterial vector, *Shigella flexneri*

## Abstract

Background: Live attenuated bacteria are promising candidates for mucosal vaccine delivery due to their ability to elicit robust immune responses. FimH is the adhesion protein of type 1 fimbriae, which is used as mucosal adjuvants. This study aims to develop a novel attenuated live bacterial vector via fimbriae recovery on *Shigella flexneri.* Methods: We generated pBAD-Fim/FWL01 by deleting IS elements in the fimbrial cluster of *S. flexneri* 2a strain T32. Transmission electron microscopy (TEM) and a mannose–sensitive agglutination assay were used to confirm that type 1 fimbriae were displayed on the recombinant strain. We then evaluated the immune induction of pBAD-Fim/FWL01 in J774A.1 murine macrophages and mice. Additionally, we used pBAD-Fim/FWL01 to deliver the neutrophil–activating protein A subunit (NapA) to assess immunogenicity. Results: Functional type 1 fimbriae on pBAD-Fim/FWL01 were confirmed using TEM and mannose–sensitive agglutination assays. Transcriptome analysis, qRT-PCR, and ELISA assays revealed that pBAD-Fim/FWL01 significantly stimulated mouse macrophages to release cytokines IL-1α, IL-1β, IL-6, and IL-10, inducing an immune response. Orally administrated pBAD-Fim-trc-napA-His/FWL01 elicited significant mucosal and humoral immune responses. Conclusions: The strain pBAD-Fim/FWL01, which expresses type 1 fimbriae, holds promise for development as an attenuated bacterial vaccine vehicle.

## 1. Introduction

Mucosal surfaces are critical interfaces for both pathogen invasion and immune defense, particularly against pathogens like *Helicobacter pylori*, *Coxsacki evirus* B3, *Vibrio cholerae*, influenza, and rotavirus. Compared to systemic vaccines, oral mucosal vaccines offer several advantages, such as convenience, cost-effectiveness, and a reduced risk of blood-borne diseases due to their needle-free delivery. However, the gastrointestinal tract presents many significant challenges to the oral delivery of vaccines, including degradation, poor delivery through mucosal barriers, and the induction of tolerance. Attenuated live bacteria have the potential to serve as vehicles for mucosal vaccine delivery. For example, attenuated *Shigella* spp. has delivered heterologous antigens, thereby eliciting immune protection against pathogenic infections [1,2,3].

Mucosal surfaces are critical interfaces for both pathogen invasion and immune defense, particularly against pathogens like *Escherichia coli* and *Salmonella* spp. Numerous studies have shown that purified FimH can bind to TLR4 directly and induce the expression of proinflammatory cytokines in macrophages and NK cells [4,5]. Microfold cells (M cells), localized in Peyer’s patches (PPs), are crucial for triggering antigen-specific mucosal immune responses due to their unique features, such as a lack of apical microvilli and a thin glycocalyx. FimH facilitates the entry of *E. coli* and *Salmonella typhimurium* into PPs by binding to glycoprotein 2 (GP2), which is specifically expressed on the apical surface of M cells [6]. Therefore, FimH has been investigated as an adjuvant for the development of oral mucosal vaccines [7,8].

*Shigella flexneri* 2a strain T32 is a spontaneous mutant characterized by the deletion of the *ipaBCDA*, *invA,* and *virG* genes, and has been demonstrated to be both safe and effective according to multiple studies [9]. FWL01, a derivative of *S. flexneri* 2a strain T32, has been used as an oral mucosal bacterial vehicle for the delivery of *S. sonnei* O antigen and *H. pylori* UreB-HspA [9,10]. IS elements are present in the *fimD* gene in 41 *S. flexneri* type 2a strains, including 2457T and Sf 301. These IS elements might appear in the fimbriae gene cluster of *S. flexneri* 2a strain T32 and affect fimbriae production [11]. In this study, we confirmed more IS elements in the fimbriae gene cluster of *S. flexneri* 2a strain T32 compared to strain 2457T, and observed the absence of fimbriae in *S. flexneri* 2a strain T32 derivative FWL01. FWL01 was capable of promoting cytokine secretion and stimulating the immune response when type 1 fimbriae were expressed on FWL01. Neutrophil-activating protein A subunit (NapA) was a considered virulence factor due to its ability to attract and activate neutrophils, bind to gastric mucus, and promote gastric inflammation [12]. NapA has been employed as a protective antigen against *H. pylori* [13].

In this work, the immunogenicity of NapA, delivered by FWL01 with surface-displayed type 1 fimbriae, was evaluated. FWL01 with type 1 fimbriae of *S. flexneri* 2a strain T32 has been shown to activate the immune response in murine macrophage cells and evoke a significant mucosal and humoral immune response.

## 2. Materials and Methods

### 2.1. Plasmid Construction

We listed the plasmids and primers in Tables S1 and S2. The fragments *fim1*-*93*, *fim1365*-*3836*, and *fim4613*-*8639* were cloned from the genome of FWL01. The *fim* gene fragment was obtained by overlap extension PCR using the above fragments and subsequently subcloned into the pBAD/My-HisA vector, resulting in the construct named pBAD-Fim. The *napA*-*His* gene was cloned from *H. pylori* SS1 genomic DNA and inserted into XbaI/PstI-digested pTrc99A, generating the construct pTrc99A-napA-His. The *trc*-*napA*-*His* gene was amplified from pTrc99A-napA-His using the primers described in Table S2 and subcloned into PaeI-digested pBAD-Fim using a Seamless Assembly Cloning Kit (CloneSmarter technologies, San Jose, CA, USA), thereby generating the recombinant vector pBAD-Fim-trc-napA-His.

### 2.2. Strains and Growth Conditions

*E. coli* strain DH5α was cultured aerobically in LB broth or on agar containing 100 μg/mL ampicillin at 37 °C for cloning. To obtain heterologous protein and extract DNA, FWL01 and its derivatives were cultured in LB broth with 50 μg/mL 2,6-diaminopimelic acid (DAP) and/or 100 μg/mL ampicillin. TSB broth containing 0.4% bile salts and 50 μg/mL DAP was employed to mimic in vivo-like conditions (IVLC) for fimbriae expression in *S. flexneri* [14]. *H. pylori* SS1 was cultured with the medium and conditions previously described in [10] for DNA extraction. *S. flexneri* 2a strain T32 was cultured in LB liquid medium for whole-genome sequencing (Majorbio, Shanghai, China).

### 2.3. Transmission Electron Microscopy (TEM) Analyses

The pBAD-Fim plasmid was transformed into FWL01, and the resulting recombinant strain was cultured overnight in LB broth (100 μg/mL ampicillin and 50 μg/mL DAP) at 37 °C. The next day, the cells were induced with 0.2% arabinose for 4 h at 37 °C. FWL01 was cultured in LB liquid medium (50 μg/mL DAP) or TSB broth (0.4% bile salts and 50 μg/mL DAP) without shaking for 3 days at 37 °C. Samples were fixed in 4% paraformaldehyde, stained with uranyl acetate, and prepared for TEM imaging using a Hitachi HT7700 microscope.

### 2.4. Mannose-Sensitive Hemagglutination Assay (MSHA) and Yeast Cell Aggregation

Functional type 1 fimbriae were evaluated using the MSHA, as described in [11]. Briefly, a bacterial suspension with an OD_600 nm_ of 4 and 2% mouse erythrocytes were mixed. The mixtures were then supplemented with 0.2 mM mannose or not. Subsequently, agglutination was monitored after agitation for 20 min.

A yeast cell aggregation assay was employed to evaluate the type 1 fimbriated bacteria [15]. Yeast cells were suspended at a concentration of 1% and mixed with recombinant strains with 0.2 mM mannose or not. Aggregation was monitored after the suspensions were mixed for 15 min.

### 2.5. Cell Culture

DMEM with 10% fetal bovine serum was used to culture mouse macrophage J774A.1 cells. Either pBAD-Fim/FWL01 or FWL01-infected J774A.1 cells were used at a multiplicity of infection (MOI) of about 20 per cell. At the times indicated after infection, J774A.1 cells were treated with TRIzol reagent (Invitrogen, Carlsbad, CA, USA) for RNA extraction and subsequently used for transcriptome analysis (Majorbio, Shanghai, China). The secretion of cytokines IL-1α, IL-1β, IL-5, IL-6, IL-10, and IFN-γ in the supernatants was quantified using an ELISA kit (CSB-E04621m; CSB-E08054m-IS; CSB-E04637m; CSB-4639m-IS; CSB-594m-IS; CSB-E04578m-IS; Cusabio, Wuhan, China).

### 2.6. Quantitative Reverse Transcriptase–Polymerase Chain Reaction (qRT-PCR)

The HiScript II Q RT SuperMix for qPCR kit was used to synthesize the cDNA (Vazyme, Nanjing, China). For transcript quantification, the AceQ qPCR SYBR Green Master Mix kit was utilized with the primers shown in Table S2. The 2^−ΔΔCT^ approach was performed to quantify the expression levels, with the GAPDH gene serving as an internal control.

### 2.7. Protein Expression and Analysis

The recombinant strains pBAD–Fim–trc–napA–His/FWL01 and pTrc99A–napA–His/FWL01 were cultured at 37 °C in LB broth supplemented with appropriate antibiotics. After overnight incubation, the cultures were inoculated and grown for an additional 3 h at 37 °C. The expression of recombinant protein was induced with arabinose (0.2%) and IPTG (1mM) for 4 h at 37 °C. Bacterial cells were harvested at an OD_600nm_ of 1 and boiled in 100 μL SDS sample buffer. Each sample was analyzed using SDS–PAGE. The expression levels of NapA–His in the recombinant strains were quantified using ImageJ 1.8.0 software.

For Western blot analysis, anti–His tag rabbit monoclonal antibody (1:3000 dilution; Easybio, Beijing, China) and HRP–conjugated goat anti–mouse IgG (1:5000 dilution; Abcam, Cambridge, UK) were used as the primary antibody and the second antibody, respectively.

### 2.8. Immunization and Antigen-Specific Antibody Determined by ELISA

Female, 6-to-8-week-old BALB/c mice (Vital River Laboratory, Beijing, China) were randomly divided into five groups. Each group was immunized on days 0, 14, and 28. Group A was immunized orally with pBAD-Fim/FWL01 at a dose of 10⁹ CFU per immunization. In group B, the mice were orally immunized at a dose of 10⁹ CFU FWL01 per immunization. Group C was orally administered pBAD-Fim-trc-napA-His/FWL01 at 10⁹ CFU per immunization. Group D received pTrc99A-napA-His/FWL01, which expressed NapA-His at levels equivalent to Group C. Group E, the control group, was administered 200 uL PBS orally for all three immunizations. The levels of antigen-specific sIgA in feces samples and IgG in serum samples were measured as previously described [16].

The Animal Care and Use Committee of the Academy of Military Medical Sciences granted approval for all animal experiments (Approval Code: IACUC-DWZX-2023-022).

### 2.9. Statistical Analysis

GraphPad Prism 8.0.2 was used to analyze the data. Statistical significance was determined using one-way ANOVA and *t*-tests, with a *p*-value < 0.05 indicating a significant difference.

## 3. Results

### 3.1. Construction of a FWL01 Derivative Strain Producing Functional Type 1 Fimbriae

**Figure 1:**
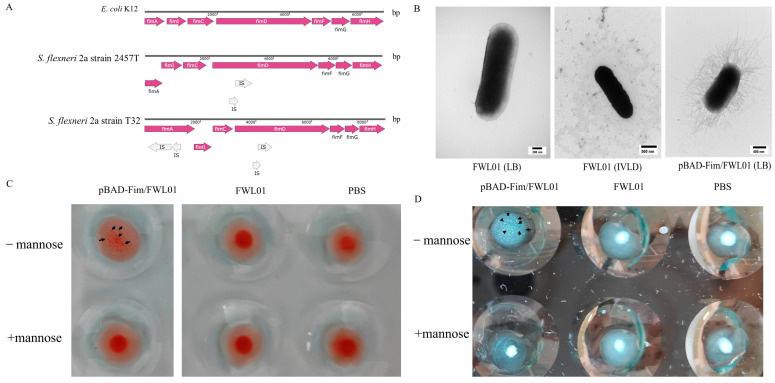
Construction of an FWL01 derivative strain displaying type 1 fimbriae. (**A**) A schematic representation of the main genes in the fimbriae cluster is shown for *E. coli* K12 (U00096.3), *S. flexneri* 2a strain 2457T (AE014073.1), and *S. flexneri* 2a strain T32. (**B**) TEM analysis of FWL01 and pBAD-Fim/FWL01 cultured in different media. (**C**) Results of mannose–sensitive agglutination of mouse erythrocytes with pBAD-Fim/FWL01 and FWL01 at OD_600 nm_ = 4. Each sample was repeated twice, and only one representative picture was selected. (**D**) Results of mannose–sensitive agglutination of yeast cells with pBAD-Fim/FWL01 and FWL01 at OD_600 nm_ = 1. Each sample was repeated twice, and only one representative picture was selected.

By aligning the fimbriae gene cluster of *S. flexneri* 2a strain T32 (Table S3) with those of *E. coli* K12 (U00096.3) and *S. flexneri* 2a strain 2457T (AE014073.1), four IS elements in fimA and fimD of the *S. flexneri* 2a strain T32 fimbriae gene cluster (Figure 1A) were identified. TEM displayed the absence of fimbriae on the surface of the FWL01 strain when cultured in either LB or the IVLC medium (Figure 1B). This absence of fimbriae in FWL01 might be due to the disturbance of FimA and FimD formation by IS elements.

In contrast, Figure 1B illustrates the presence of fimbriae on the recombinant strain pBAD-Fim/FWL01, suggesting that fimbriae were recovered following the expression of a fimbriae gene cluster with the IS elements knocked out.

Type 1 fimbriae expression was validated using a MSHA and yeast cell aggregation assays. Eukaryotic cells are rich in mannose receptors, which can bind to type 1 fimbriae. The mixture appears cloudy or granular, indicating cell agglutination. The agglutination disappears upon the addition of mannose, which serves as the standard criterion for identifying type 1 fimbriae. As shown in Figure 1C,D, pBAD-Fim/FWL01 exhibited agglutination in the absence of mannose, while no agglutination was observed with mannose. These data suggest that pBAD-Fim/FWL01 successfully expressed functional type 1 fimbriae.

### 3.2. Transcriptome Analysis of J774 A.1 Cells Treated with FWL01 Expressing Type 1 Fimbriae of S. flexneri 2a T32

**Figure 2:**
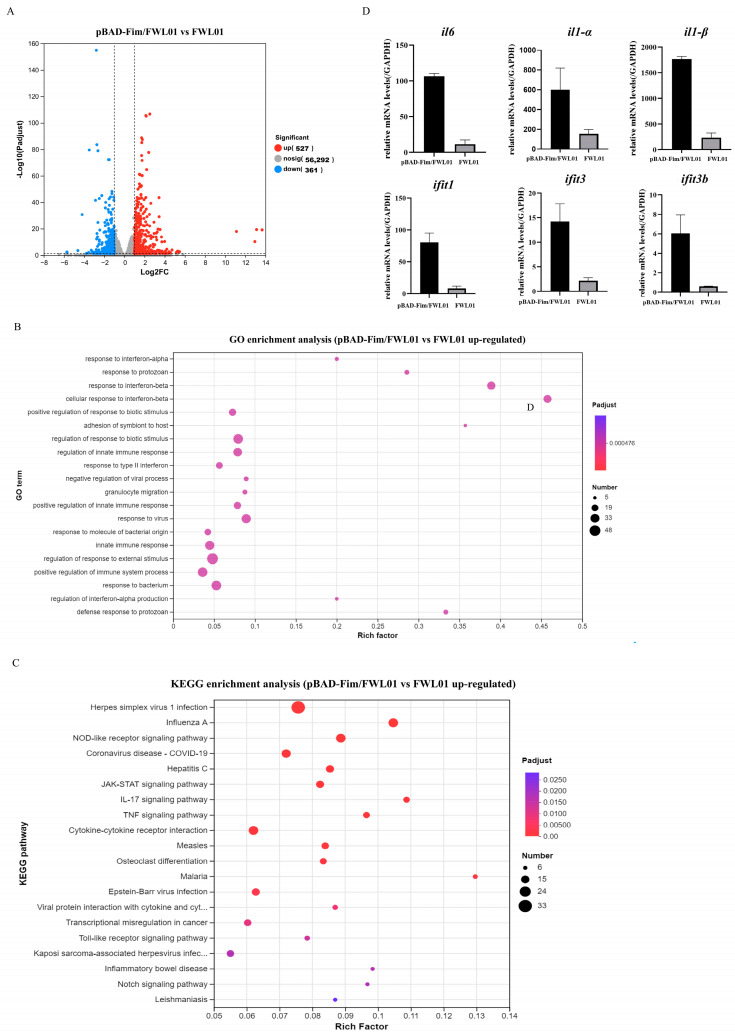
Transcriptome analysis and RNA sequencing validation. (**A**) Volcanic map analysis of transcriptomic differentially expressed genes of mouse macrophage J774A.1 cells treated with pBAD-Fim/FWL01 and FWL01, respectively. Red dots on the right represent upregulated genes, while blue dots on the left denote downregulated genes. (**B**) Top 20 GO enrichment pathways of upregulated genes. (**C**) Top 20 KEGG enrichment pathways of upregulated genes. (**D**) The expression levels of *il6*, *il1α*, *il1β*, *ifit1*, *ifit3*, and *ifit3b* were upregulated in J774A.1 cells infected with pBAD-Fim/FWL01 for 3 h (MOI = 20), as determined by qRT-PCR.

Further, we investigated the transcriptomic profiles of J774 A.1 cells treated with either pBAD-Fim/FWL01 or FWL01 (Figure 2). A comparative analysis revealed 888 differentially expressed genes (DEGs) (|log2FC| ≥ 1 and *p* < 0.05) between the pBAD-Fim/FWL01-treated group and the FWL01-treated group; 527 genes were up-regulated (higher expression in pBAD-Fim/FWL01-treated group), while 361 genes were downregulated. Gene Ontology (GO) enrichment analysis revealed the significant enrichment of 812 biological process terms, 56 cellular component terms, and 241 molecular function terms among the upregulated DEGs. The top 20 GO terms were predominantly associated with BP, including response to interferon-α, response to interferon-β, and the regulation of innate immune response. Additionally, KEGG pathway enrichment analysis identified Toll-like receptor signaling as one of the most significantly enriched pathways among the upregulated DEGs. We analyzed the expression levels of several upregulated genes by qRT-PCR. Consistent with our expectations, the qRT-PCR results basically corroborated the RNA-seq results (Figure 2D).

### 3.3. Type 1 Fimbriae of S. flexneri 2a Stimulated the Cytokine Secretion of J774 A.1 Cells and Induced Antigen-Specific Antibodies

**Figure 3:**
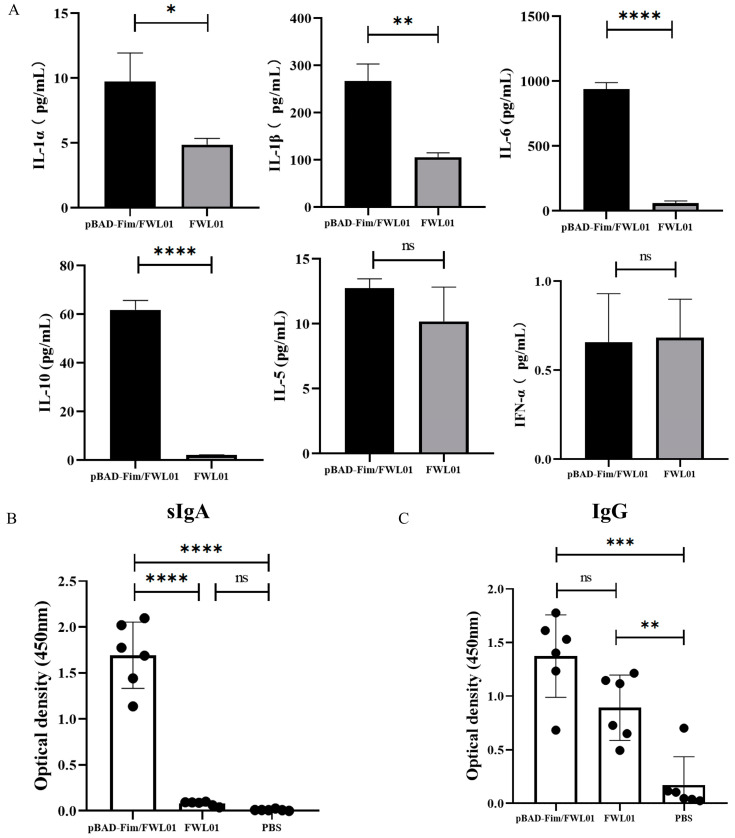
Type 1 fimbriae of *S. flexneri* 2a stimulated cytokine secretion by mouse macrophage J774A.1 cells and induced antigen-specific antibodies. (**A**) pBAD-Fim/FWL01 and FWL01-infected mouse macrophages at MOI = 1:20. ELISA was performed to confirm cytokine secretion. (**B**) Group A and B mice were immunized orally with 10^9^ CFU pBAD-Fim/FWL01 and FWL01 on days 0, 14, and 28, respectively; Group E mice were immunized with PBS. The ELISA plates were coated with pBAD-Fim/FWL01 debris (100 μL, 4 μg/mL). Feces and serum were collected from mice on day 38, and antigen-specific sIgA (1:8 dilution) (**B**) and IgG (1: 100 dilution) (**C**) were determined by ELISA. **** *p* < 0.0001, ***< *p* < 0.001, ** *p* < 0.01, * *p* < 0.05 and ns *p* > 0.05.

Following the stimulation of J774 A.1 cells, pBAD-Fim/FWL01 resulted in an elevated secretion of IL-1α, IL-1β, IL-6, and IL-10 by 1.99-, 2.5-, 15.6- and 28.32-fold, respectively, compared to that of FWL01 (Figure 3A). However, there were no significant differences in the secretion of IL-5 and IFN-γ in J774A.1 cells stimulated with pBAD-Fim/FWL01 and FWL01.

Following immunization with 10^9^ CFU pBAD-Fim/FWL01 or FWL01, the levels of secretory IgA (sIgA) in the feces and IgG in the serum of immunized mice were examined using ELISA. As shown in Figure 3B, mice immunized with pBAD-Fim/FWL01 exhibited significantly elevated levels of antigen-specific sIgAs compared to those immunized with FWL01. Both the pBAD-Fim/FWL01-treated group and the FWL01-treated group produced significantly higher levels of antigen-specific IgGs compared to the PBS-treated group. No significant difference was observed between the pBAD-Fim/FWL01-treated group and the FWL01-treated group.

These findings indicate that restoring the expression of fimbriae in FWL01 may enhance mucosal immune responses.

### 3.4. Expression of Heterologous Proteins

**Figure 4:**
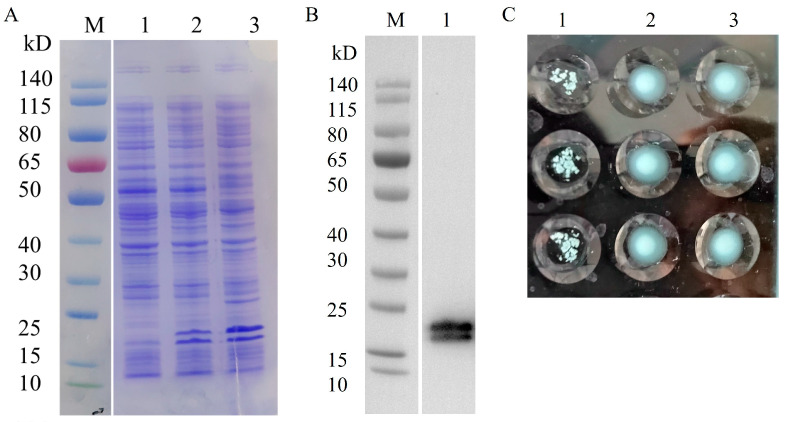
Co-expression of NapA and type 1 fimbriae in pBAD-Fim-trc-napA-His/FWL01. (**A**) SDS-PAGE displayed that NapA-His was expressed in the recombinant strains. Lane 1, FWL01; Lane 2, pTrc99A-napA-His/FWL01; Lane3, pBAD-Fim-trc-napA-His/FWL01. (**B**) Western blot analysis of heterologous proteins expressed in pBAD-Fim-trc-napA-His/FWL01 using antibodies against His-tag. Lane1, pBAD-Fim-trc-napA-His/FWL01. (**C**) Results of agglutination assay of yeast cells at OD_600 nm_ = 4. Lane1, pBAD-Fim-trc-napA-His/FWL01; Lane 2, pTrc99A-napA-His/FWL01; Lane 3, PBS. Three replicates were performed in each group.

The expression of heterologous proteins in recombinant strains was induced using IPTG. pBAD-Fim-trc-napA-His/FWL01 and pTrc99A-napA-His/FWL01 produced NapA-His successfully with the predicted molecular weight of 18 kDa, according to SDS-PAGE and Western blot analysis. The presence of an additional band may indicate a degradation product (Figure 4A,B). In comparison to pTrc99A-napA-His/FWL01 and PBS, the induced pBAD-Fim-trc-napA-His/FWL01 demonstrated yeast cell agglutination (Figure 4C), suggesting type 1 fimbriae were displayed on the surface of the pBAD-Fim-trc-napA-His/FWL01 strain.

### 3.5. Immunization and Detection of Antigen-Specific Antibodies

**Figure 5:**
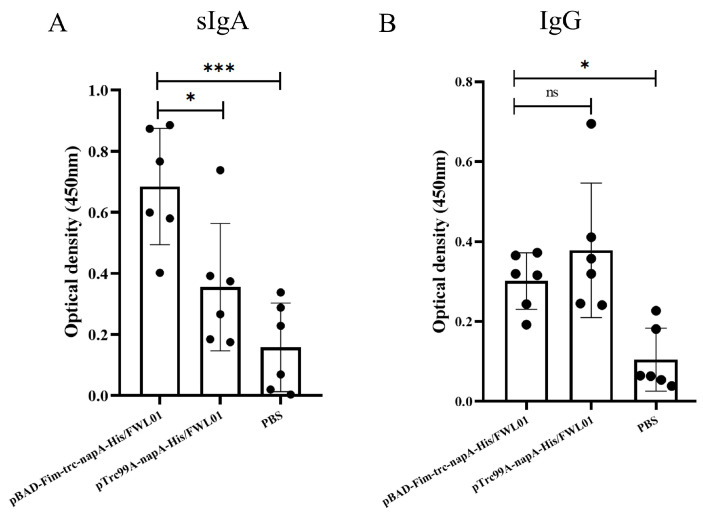
Immune response induced by immunization. Mice in groups C and D received oral administrations of pBAD-Fim-trc-napA-His/FWL01 and pTrc99A-napA-His/FWL01 on days 0, 14, and 28, respectively. Mice in group E were immunized orally with PBS. Feces and serum were collected from mice on day 38. NapA-specific sIgA (1:2 dilution) (**A**) and IgG (1:100 dilution) (**B**) levels were determined. *** *p* < 0.001, * *p* < 0.05, and ns *p* > 0.05.

Mice were orally immunized with pBAD-Fim-trc-napA-His/FWL01 and pTrc99A-napA-His/FWL01. The mucosal and humoral immune responses elicited by this immunization were subsequently assessed. In comparison to the control group (E), both groups C and D exhibited the production of antigen-specific mucosal sIgAs. As illustrated in Figure 5A, the level of antigen-specific sIgAs in group C was significantly elevated compared to that in group D. Antigen-specific IgG levels in groups C and D were significantly higher than in group E. Meanwhile, the levels of serum antigen-specific IgG were not significantly different between group C and group D (Figure 5B).

## 4. Discussion

Fimbriae play a crucial role in the adhesion of bacteria to mammalian cells within the Enterobacteriaceae family. The type 1 fimbriae gene cluster consists of nine genes. *S. flexneri* strains are generally non-fimbriated [15]. Chanin et al. reported that the presence of bile salts and glucose promotes the formation of type 1 fimbriae in *Shigella* spp., facilitating biofilm formation and attachment to colonic epithelial cells [14]. However, TEM revealed that the FWL01 strain did not exhibit fimbriae when cultured in IVLC medium in our study. Upon knocking out the insertion sequence (IS) elements from the fimbriae gene cluster of FWL01, fimbriae became apparent on the surface of FWL01. The MSHA and the agglutination assay of yeast cells demonstrated that the fimbriae of *S. flexneri* 2a strain T32 were of the type 1 variety.

FimH, located at the tip of type 1 fimbriae, mediates bacterial adhesion to mucosal surfaces, facilitating colonization and immune modulation. FimH from *E. coli* or *Salmonella* spp. was reported as a novel TLR4 ligand to elicit the secretion of pro-inflammatory cytokines, thereby providing potent signals to induce both adaptive and innate immune responses. For example, Uchiya et al. reported that FimH from *Salmonella* spp. could induce TLR4-mediated IL-1β expression. The expression levels of both IL-6 and TNF-α mRNAs were significantly upregulated, as measured by qRT-PCR [4]. Mossman et al. reported that FimH from *E. coli* could induce innate responses via interaction with TLR4 independently of LPS. The secretion of TNF-α in peritoneal macrophages induced by FimH from *E. coli* increased significantly [5]. Liu et al. reported that purified FimH stimulated the maturation of DC2.4 and provoked cytokine secretion, including IL-6, IL-10, and TNF-α, through the TLR-dependent signaling pathway. Then, it was reported that mice immunized with FimH and PAc could produce remarkable PAc-specific IgG in serum and sIgA in saliva and evoke splenocyte proliferation [17]. Through sequence alignment, the FimH from *S. flexneri* 2a strain T32 showed high identity at the amino acid level with FimH from uropathogenic *E. coli*. Therefore, we speculated that fimH from *S. flexneri* 2a strain T32 might induce a similar immune response to FimH from *E. coli*. In this study, we indeed found that higher levels of IL-1α, IL-1β, IL-6, and IL-10 were secreted in J774A.1 cells treated with pBAD-Fim/FWL01 than in J774A.1 cells treated with FWL01.

IL-1β is a key cytokine involved in regulating cellular proliferation, differentiation, and apoptotic pathways. Additionally, IL-1 is known to facilitate the maturation of dendritic cells (DCs) and enhance the responses of CD4+ and CD8+ T cells [18]. IL-10 can promote B cells to switch antibody production to IgA and function as an anti-inflammatory and regulatory cytokine, thereby modulating initial inflammatory responses. Cytokines, including IL-10, IL-6, and transforming growth factor-β, are crucial in the synthesis and maintenance of IgA at mucosal surfaces [19,20]. In this study, mice immunized with pBAD-Fim/FWL01 evoked more antigen-specific sIgAs than mice immunized with FWL01. Then, we used the pBAD-Fim/FWL01 strain to deliver NapA to immune mice. The level of NapA-specific sIgA in the pBAD-Fim-trc-napA-His/FWL01 group was higher than that in the pTrc99A-napA-His/FWL01 group.

However, we found there was no difference between the pBAD-Fim-trc-napA-His/FWL01 group and the control group in the levels of NapA-specific spleen CD4+ T cells and cytokine-secreted spleen CD4+ T cells after the third immunization, as established by flow cytometric analyses. Next, we would study peyer’s patches and lamina propria lymphocytes o explore the mechanism of mucosal immunity induced by the recombinant strain.

In this study, the levels of NapA-specific sIgA and IgG induced by pBAD-Fim-trc-napA-His/FWL01 were not substantial, potentially due to the limitation of antigen expression. Future efforts will focus on improving the expression level and the presentation of antigens to increase the production of antigen-specific antibodies.

## 5. Conclusions

In summary, the IS elements within the fimbriae gene cluster disturbed type 1 fimbriae formation in *Shigella* spp. Type 1 fimbriae of *S. flexneri* 2a strain T32 have been shown to activate the immune response in murine macrophage cells. Furthermore, the delivery of NapA using FWL01 with displayed type 1 fimbriae on its surface as a live bacterial vehicle elicits a significant mucosal and humoral immune response. The FWL01 strain expressing type 1 fimbriae holds potential for development as an attenuated bacterial vaccine vehicle.

## Data Availability

Data are contained within the article and Supplementary Materials. Further inquiries can be directed to the corresponding authors.

## Supplementary Materials

The following supporting information can be downloaded at: https://www.mdpi.com/article/10.3390/vaccines13030280/s1, Table S1: Bacterial strains and plasmids; Table S2: Oligonucleotides used in this study; Table S3: The fimbriae gene cluster sequences of *S. flexneri* 2a strain T32 used in this study.

## References

[1] Yan X., Wang D., Liang F., Fu L., Guo C. (2014). HPV16L1-attenuated *Shigella* recombinant vaccine induced strong vaginal and systemic immune responses in guinea pig model. Hum. Vaccin. Immunother..

[2] Pilla G., Wu T., Grassel C., Moon J., Foulke-Abel J., Tang C.M., Barry E.M. (2021). Evaluation of a Live Attenuated *S. sonnei* Vaccine Strain in the-HumanEnteroid Model. Pathogens.

[3] Xu D., Wang D., Yang X., Cao M., Yu J., Wang Y. (2014). Fusion of HPV L1 into *Shigella* surface IcsA: A new approach in developing live attenuated Shigella-HPV vaccine. Antiviral Res..

[4] Uchiya K.I., Kamimura Y., Jusakon A., Nikai T. (2019). *Salmonella* fimbrial protein FimH Is involved in expression of proinflammatory cytokines in a toll-like receptor 4-dependent manner. Infect. Immun..

[5] Mossman K.L., Mian M.F., Lauzon N.M., Gyles C.L., Lichty B., Mackenzie R., Gill N., Ashkar A.A. (2008). Cutting Edge: FimH Adhesin of Type 1 Fimbriae Is a Novel TLR4 Ligand. J. Immunol..

[6] Hase K., Kawano K., Nochi T., Pontes G.S., Fukuda S., Ebisawa M., Kadokura K., Tobe T., Fujimura Y., Kawano S. (2009). Uptake through glycoprotein 2 of FimH^+^ bacteria by M cells initiates mucosal immune response. Nature.

[7] Vilander A.C., Shelton K., LaVoy A., Dean G.A. (2023). Expression of *E. coli* FimH enhances trafficking of an orally delivered *Lactobacillus acidophilus* vaccine to immune inductive sites via antige-presenting cells. Vaccines.

[8] Fan X., Yue Y., Xiong S. (2017). Incorporation of a bi-functional protein FimH enhances the immunoprotection of chitosan-pVP1 vaccine against coxsackievirus B3-induced myocarditis. Antivir. Res..

[9] Wang H., Feng E., Lin Y., Liao X., Jin M., Jin M., Huang L., Su G., Huang C. (2002). Construction of a trivalent candidate vaccine against *Shigella* species with DNA recombination. Sci. China.

[10] Zhang X., Sang S., Guan Q., Tao H., Wang Y., Liu C. (2022). Oral administration of a Shigella 2aT32-Based vaccine expressing UreB-HspA fusion antigen with and without parenteral rUreB-HspA boost confers protection against *Helicobacter pylori* in mice model. Front. Immunol..

[11] Bravo V., Puhar A., Sansonetti P., Parsot C., Toro C.S. (2015). Distinct mutations led to inactivation of type 1 fimbriae expression in *Shigella* spp.. PLoS ONE.

[12] Fu H.W. (2014). *Helicobacter pylori* neutrophil-activating protein: From molecular pathogenesis to clinical applications. World J. Gastroenterol..

[13] Peng X., Zhang R., Duan G., Wang C., Sun N., Zhang L., Chen S., Fan Q., Xi Y. (2018). Production and delivery of *Helicobacter pylori* NapA in *Lactococcus lactis* and its protective efficacy and immune modulatory activity. Sci. Rep..

[14] Chanin R.B., Nickerson K.P., Llanos-Chea A., Sistrunk J.R., Rasko D.A., Kumar D.K.V., de la Parra J., Auclair J.R., Ding J., Li K. (2019). *Shigella flexneri* adherence factor expression in in vivo-like conditions. mSphere.

[15] Gbarah A., Mirelman D., Sansonetti P.J., Verdon R., Bernhard W., Sharon N. (1993). *Shigella flexneri* transformants expressing type 1 (mannose specific) fimbriae bind to, activate, and are killed by phagocytic cells. Infect. Immun..

[16] Sang S., Song W., Lu L., Ou Q., Guan Y., Tao H., Wang Y., Liu C. (2024). The trimeric autotransporter adhesin SadA from *Salmonella spp.* as a novel bacterial surface display system. Vaccines.

[17] Liu Z.F., Chen J.L., Li W.Y., Fan M.W., Li Y.H. (2019). FimH as a mucosal adjuvant enhances persistent antibody response and protective efficacy of the anti-caries vaccine. Arch. Oral Biol..

[18] Yazdi A.S., Ghoreschi K. (2016). The Interleukin-1 Family. Adv. Exp. Med. Biol..

[19] Bagheri Y., Babaha F., Falak R., Yazdani R., Azizi G., Sadri M., Abolhassani H., Shekarabi M., Aghamohammadi A. (2019). IL-10 induces TGF-β Secretion, TGF-β receptor II Upregulation, and IgA secretion in B cells. Eur. Cytokine Netw..

[20] Kannan N., Choi A., Rivera De Jesus M.A., Wei P.M., Sahler J.M., Curley S.M., August A., DeLisa M.P., Whittaker G.R., Putnam D. (2024). Intranasal vaccination with recombinant TLR2-active outer membrane vesicles containing sequential M2e epitopes protects against lethal influenza a challenge. Vaccines.

