# Supplementary material for: Surface Display of Type 1 Fimbriae on Shigella flexneri Induces Antigen-Specific Immune Response via Oral Route
Source: Vaccines (Basel). 2025 Mar 6;13(3):280. doi: 10.3390/vaccines13030280 (PMC11945882; doi:10.3390/vaccines13030280)
Supplement: Supplementary file 1 [file vaccines-13-00280-s001.zip › vaccines-3461590-supplementary.pdf]

Table S1. Bacterial strains and plasmids.

Strain or plasmid	Genotype or description	Reference or source
Stains		
DH5α	Cloning strain	CWBIO, China
Shigella flexneri 2a strain T32	Strain for whole genome sequencing	This laboratory
FWL01	*Shigella flexneri* 2a Strain T32 *asd::ctxB*	This laboratory
H. pylori SS1	Strain for cloning *napA* gene	This laboratory
Plasmids		
pBAD/Myc−HisA	*amp*, P_{araBAD}, used for expressing the fimbriae	Lab collection
pTrc99A	*amp*, P_{trc}, used for expressing the heterologous proteins	Lab collection
pBAD−Fim	pBAD/Myc−HisA derivative containing *Shigella* fimbriae gene without IS element genes	This work
pTrc99A−napA−His	*pTrc99A* derivative containing *napA* with the gene of His−tag	This work
pBAD−Fim−trc−napA−His	pBAD/Myc−HisA derivative containing *Shigella* fimbriae gene without IS element genes, P_{trc} and *napA* with the gene of His−tag	This work

Table S2. Oligonucleotides used in this study.

Name	Sequence	Target
1−up−fim−F	CATGCCATGGTGTTAAGGCATGCTTGCGGTTATG	*fim*
1−up−fim−R	TTAAAGTGAATGGTCCCACCATTTACCGTCGTAGT	*fim*
2−up−fim−F	GGTAAATGGTGGGACCATTCACTTTAAAGGGGAAG	*fim*
2−up−fim−R	CAATCCGATTCTGTACACTATTTCCGCTGAA	*fim*
3−up−fim−F	GGAAATAGTGTACAGAATCGGATTGGGGGTAAC	*fim*
3−up−fim−R	CGCGTCGACTTATTGATAAACAAAAGTCACG	*fim*
fim−F	CATGCCATGGGCAAAATTAAAACTCTGGCAATCGTTGTTC	*fim*
fim−R	CCCAAGCTTTTATTGATAAACAAAAGTCACGCCA	*fim*
napA−His−F	CTAGTCTAGAATGAAAACATTTGAAATTTT	*napA−His*
napA−His−R	AAACTGCAGTTAATGATGATGATGATGATGACTACCACCA CCGCCAGCTAAATGGGCTTC	*napA−His*
pBAD−Fim−napA−His−F	GAAGCGGCATGCATTTACGTTGA	*trc−napA−His*
pBAD−Fim−napA−His−F	AGGCACATTATGTTAATGATGATGAT	*trc−napA−His*
il6−F	TTAGCCACTCCTTCTGTGACTCC	*il6*
il6−R	ACCCCAATTTCCAATGCTCT	*il6*
il1α−F	CAGTTCTGCCATTGACCATC	*il1α*
il1α−R	ATGGACTGCAGGTCATCTTC	*il1α*
il1β−F	AGAGCTTCAGGCAGGCAGTAT	*il1β*
il1β−R	GAAGGTGCTCATGTCCTCATC	*il1β*
ifit1−F	CCAAGTGTTCCAATGCTCCT	*ifit1*
ifit1−R	GGATGGAATTGCCTGCTAGA	*ifit1*
ifit3−F	AGACAGGGTGTGCAACCAGG	*ifit3*
ifit3−R	CGACGAATTTCTGATTGATC	*ifit3*
ifit3b−F	AGACAGGGTGTGCAACCAGC	*ifit3b*
ifit3b−R	CGGGCGAATTTCTGCTTGATC	*ifit3b*
gapdh−F	AGGTTGTCTCCTGCGACTTC	*gapdh*
gapdh−-R	ACTCCTTGGAGGCCATGTAG	*gapdh*

Table S3. The fimbriae gene cluster sequences of *S. flexneri* 2a strain T32 used in this study.

the fimbriae gene cluster sequences of *S. flexneri* 2a strain T32 (yellow, IS)	atgaaaattaaaactctggcaatcgttgttctgtcggctctgtccctcagttccgcagcggctctggccgatactacga<br>cggtaaatggtgggactgaggtgtactggcaatagcggacactaccatttgttcttttttaagcagccatctgatgat<br>atttttccctgaaggctgccggggagatattccccagacgagagtgacgacgctgacgattgtagaaaatctcaatg<br>tattcccgtattactgagatggcttcatcccggttattaaaacgatagtggctcaggctctcatttttcagcgttccccag<br>aagctttccatcggagcgttgtcgtaacagttacctttacgcgacattgatgttttcagaccaaactgctcctgtatgac<br>ccggtaatcgtatgcgcagtactgtgaacctcgatcagagtggtggattagcccggcaggtgggcgctggctcct<br>gagcgccataaacagggctttacctgtcagctcttttgtcatgcgctctcccatggcgtagccgacaatttcgcacgt<br>ataaacatctttgatgccagcgaggtacaaccatccctcctgtgtggcaacatacgtcaggtccgccacccagacct<br>gatttggtgctgtaggagcgaacgtctggttcagcagatttggcgcaactggcagattgtggttcgggttcgtagtc<br>gctctgaacttgcgtttctgcttacagcgtagcctcagctccttacgaagacgtgccagtcggtcacgaccaacgat<br>gatgccattctctgccagctccgtctggagccgccgggttccatatgtttcgcgagtgcggatatgtgccaccttaat<br>ctccagttttagccgctcatcactttgttttctgtctgagggttcatgctgtacccagttgtaataaccgctcctggatac<br>accaaatacctgacacatcgcttcaatgggaaattgttgtcgccattgttcgattaacgcgtatttttcagcgactcctg<br>tgcaaaatacgctgttgcttttttaatatatctcgctcaaggcgagcttcatttaacgccttacgcagttgcagaatttca<br>gattccagttcagccagcgtgcgggaaccaggagtaccgagcccttttctggcggcggtaacccattgtcctaaag<br>tgccttcaggaagagataatcgggaagcgccttcactgatcgaaagttgattttcaagaaccgttctgacagcttcgg<br>ctttgaactctttagagtaacgttgggtttttctgctcattattagctccttctgatgccattctatttcaggaaggagtgtc<br>cgttaaactcaggctacctcagaccattcactttaaaggggaagttgttaacgccgcttgcgcagttgatgcaggctc<br>tgttgatcaaaccgttcagttgggacaggttcgtaccgctagcctgaagcaggctggagcaaccagctctgccgtt<br>ggttttaacattcagctgaatgattgcgataccactgttgccacaaaagccgctgttgccttcttaggtacggcaattg<br>atgctacgcgtactgatgtactggctctgcagagttctgctgcaggtagtgcaacaaacgttggcgtgcagatccta<br>gacagaacaggcaatgctctgacgctggacggtgcgacatttagtgcacaaacaaccctgaataacggtaccaac<br>accattccgttccaggcgcgttattatgcaatcggcgaggcaaccccgggtgcagctaatgcggatgcaaccttca<br>aggttcagtatcaataacctacccaggttcaaggacgtcattacgggcagggatgcccacccttgtgcgataaaaat<br>aacgatgaaaaggaagagattatttctgttagcgtcgttgctgccaatgtttgctctggccggaaataaatggaatac<br>cacgctgcccggcggaaatatgcaatttcagggcgtcattattgcggaaacttgccggattgaagccggtgataaa<br>caaatgacggtcaatatggggcaaatcagcagtaaccggtttcatgcggcaggggaagatagcgcaccggtgcc<br>ttttgttattcatttacgggaatgtagcacggtggtgagtgaacgtgtgggtgtggcgtttcacggtgtcgcggatggt<br>aaaaatccggatgtgctctccgtgggagaggggccagggatagcctccaatattggtgtagcgttgtttgatgatga<br>aggaaacctcgtaccgattaatcgtcctccagcaaactggaaacggctttactcaggctctacttcgctacatttcatc<br>gccaaatatcgtgctaccgggcgtcgggttactggcggcatcgccaatgcccaggcctggttctctttaacctatca<br>gtaattgttcagtagataatgtgataacaggaacaggacagtgagtaataaaaacgtcaatgtaaggaaatcgcag<br>gaaataacattctgcttgctggcaggtatcctgatgttcatggcaatgatggttgccggacgcgctgaagcaggagt<br>ggccttaggtgcgactcgcgtaatttatccggcagggcaaaaacaagtgcaacttgccgtgacaaataatgatgaa<br>aatagtacctatttaattcaatcatgggtggaaaatgccgatggtgtaaaggatggtcgttttatcgtgacgcctcctct<br>gtttgcgatgaagggaaaaaaagagaataccttacgtattcttgatgcaacaaataaccaattgccacaggaccgg<br>gaaagtttattctggatgaacgttaaagcgattccgtcaatggataaatcaaaattgactgagaatatgctacagctcg<br>caattatcagccgcattaaactgtactatcgcccggctaaattagcgttgccacccgatcaggccgcagaaaaatta<br>agatttcgtcgtagcgcgaattctctgacgctgattaacccgacaccctattacctgacggtaacagagttgaatgcc<br>ggaacccgggttcttgaaaatgcattggtgcctccaatgggcgaaagcacggttaaattgccttctgatgcaggaa<br>gcaatattacttaccgaacaataaatgattatggcgcacttacccccaaaatgacgggcgtaatggaataacgcagg<br>gggaatttttcgcctgaataaaaagaattgactgccgggtgattttaaaccggaggaataatgtcatatctgaatttaa<br>gactttaccagcgaaacacacaatgcttgcatattcgtaagcatcgtttggctggtttttttgtccgactcgttgtcgcct

	gtgcttttgccgcacaggcacctttgtcatctgccgacctctattttaatctgcgctttttagcggatgatccccaggct<br>gtggccgatttatcgcgttttgaaaatgggcaagaattaccgccagggacgtatcgcgtcgatatctatttgaataat<br>ggttatatggcaacgcgtgatgtcacatttaatacgggcgacagtgaacaagggattgttccctgcctgacacgcgc<br>gcaactcgccagtatggggctgaatacggcttctgtcgccggtatgaatctgctggcggatgatgcctgtgtgccat<br>taaccacaatggtccaggacgctactgcgcatctggatgttggtcagcagcgactgaacctgacgatccctcaggc<br>atttatgagtaatcgcgcgcgtggttatattcctcctgagttatgggatcccggtattaatgccggattgctcaattataa<br>tttcagcggaaatagtgtacagaatcgggtaatgctgccaacttactgatttagtgtatgatggtgattttaaggtgctt<br>gcgtggcttccatttccatcagatgtccttcctgctccgctactgaaggcgtggtgcgtaacggcaaaagcactgcc<br>ggacatcagcgctatctctgctctcattgccgtaaaacatggcaactacagttcacttacaccgcctctcagcccggt<br>aagcaccagaaaatcattgatatggccatgaatggcgtcggatgtcgcgccagtgcacgcattatgggcgttggcc<br>tcaacacggttttacgtcacttaaaaaactcaggccgcagtcggtaacctcgcgcatacaaccgggcagtgatgtg<br>attgtctgcgctgaaatggacgaacattggggctacgtcggtgctaaatcacgtcagcgctggctgttttacgcgtat<br>gacaggatacggaggacggttgtggcgcacgtcttcggtgaacgcactctggccacactggagcgtcttctgagc<br>ctgctgtcggcctttgaggtcgtggtatggatgacggatggctgtccgctgtatgaatcacgcctgaagggaaagct<br>gcacgttatcagcaagcgttacactcagcgcattgagcgacataatctgaatctgagacaacatctggcaaggctg<br>gtacggaagtcactgtcgttctcaaaatcggtggagctgcatgacaaggtcatcgggcattatctgaacataaaaca<br>ctatcagtaagttggagtcattacccagaatcggattgggggtaacagccattatgcatatttaaacctacagagtgg<br>gttaaatattggtgcgtggcgtttacgcgacaataccacctggagttataacagtagcgacagttcatcaggtagcaa<br>aaataaatggcagcatatcattacctggattgagcgagacataattcctttacgttcccggctgacgttgggtgatggt<br>tatacccagggcgatattttcgatggtattaactttcgcggcgcacaattggcctcagatgacaatatgttacccgata<br>gccaaagaggatttgccccggtgatccacggtattgctcgtggtactgcacaggtcactattaaacaaaatggatat<br>ggcatttataatagtacggtgccaccggggccttttaccatcaacgatatctatgccgcaggtaatagtggtgacttg<br>caggtaacgatcaaagaggctgacggcagcacgcagatttttaccgtaccctattcgtcagtcccgcttttgcaacg<br>tgaagggcatactcgttattccattacggcaggagaataccgtagtggaaatgcgcagcaggaaaaaccccgcttt<br>ttccagagtacattactccacggccttccggctggctggacaatatatggtggaacgcaactggcggatcgttatag<br>tgcttttaatttcggtatcgggaaaaacatggaggcactgggcgctctgtctgtggatatgacgcaggctaattccac<br>acttcccgatgacagtcagcatgacggacaatcggtgcgttttctctataacaaatcgctcaatgaatcaggcacga<br>atattcagttagtgggttaccgttattcgaccagcggatattttaatttcgctgatacaacatacagtcgaatgaatggc<br>tacaacatcgaaacacaggatggagttattcaggttaagccgaaattcaccgactattacaacctcgcttataacaaa<br>cgcgggaaattacagctcaccgttactcagcaactcgggcgcacatcaacactgtatttgagtggtagccatcaaa<br>cttattggggaacgagtaatgtcgatgagcaattccaggctggattaaatactgcgttcgaagatatcaactggacg<br>ctcagctatagcctgacgaaaaacgcctggcaaaaaggacgggatcagatgttagcgcttaacgtcaatattccttt<br>cagccactggctgcgttctgacagtaaatctcagtggcgacatgccagtgccagctacagcatgtcacacgatctc<br>aacggtcggatgaccaatctggctggtgtatacggtacgttgctggaagacaacaacctcagttatagcgtgcaaa<br>ccggctatgccgggggaggcgatggtaatagcggaagtacaggctacgccacgctgaattatcgcggtggttac<br>ggcaatgccaatatcggttacagccatagcgatgatattaagcagctctattacggagtcagcggtggggtattggc<br>tcatgccaatggcgtaacgctggggcagccgttaaacgatacggtggtgcttgttaaagcgcctggcgcaaaaga<br>tgcaaaagtcgaaaaccagacgggggtgcgtaccgactggcgtggttatgccgtgctgccttatgccactgaatat<br>cgggaaaatagagtggcgctggataccaataccttggctgataacgtcgatttagataacgcggtcgctaacgttgt<br>tcccactcgtggggcgatcgtgcgagcagagtttaaagcgcgcgttgggataaaactgctcatgacgctaactcac<br>aataataagccgctgccgtttggggcgatggtgacatcagagagtagccagagtagcggcattgttgcggataatg<br>gtcaggtttacctcagcggaatgcctttagcgggaaaagttcaggtgaaatggggagaagaggaaaatgctcact<br>gtgtcgccaattatcaactgccaccagagagtcagcagcagttattaacccagctatcagctgaatgtcgttaaggg<br>ggcgtgatgagaaacaaaccttttatcttctgtgcgctttttgtggctggcggtaagtcgcgttttggctgcggatag

	cacgattactatccgcggctatgtcagagataacggctgtagtgtggccgctgaatcaaccaattttactgttgatctg<br>atggaaaacgcggcgaagcaatttaacaacattggcgcgacgactcctgtcgttccatttcgtattttgctgtcaccct<br>gtggtaatgccgtttctgccgtaaaagttgggtttaccggcgttgcagatagccacaatgccaacctgcttgcacttg<br>aaaatacggtgtcagcggctgcgggactgggaatacagcttctgaatgagcagcaaaatcaaataccccttaatgc<br>tccatcgtctgcgatttcgtggacgaccctgacgccgggtaaaccaaatacgctgaatttttacgcccggctaatgg<br>cgacacaggtgcctgtcactgcggggcatatcaatgctacggctaccttcactcttgaatatcagtaactggagatg<br>ctcatgaaatggtgcaaacgtgggtatgtattggcggcaatgttggcgctcgcaagtgcgacgatacaggcagcc<br>gatgtcaccatcacggtgaacggtaaggtcgtcgccaaaccgtgtacggtttccaccaccaatgccacggtagatc<br>tcggcgatctttattctttcagtcttatgtctgccggggcggcatcggcctggcatgatgttgcgcttgagttgactaat<br>tgtccggtgggaacgtcgagggtcactgccagcttcagcggggcagccgacagcaccggatattataaaaacca<br>ggggaccgcgcaaaacatccagttagagctacaggatgacagtggcaacacattgaatactggcgcaaccaaaa<br>cagttcaggtggatgattcctcacaatcagcgcacttcccgttacaggtcagagcattgacggtaaatggcggagc<br>cactcagggaaccattcaggcagtgattagcatcacctatacctacagctgaacccgaagagataattgtaatgaaa<br>cgagctattaccctgtttgctgtactgctgatgggctggtcggtaaatgcctggtcattcgcctgtaaaaccgccaat<br>ggtaccgctatccctattggcggtggcagcgctaatgtttatgtaaaccttgcgcctgtcgtgaatgtggggcaaaa<br>cctggtcgtagatctttcgacgcaaatcttttgccataacgattatccggaaaccattacagactatgtcacactgcaa<br>cgaggctcggcttacggcggcgtgttatctaatttttccgggaccgtaaaatatagtggcagtagctatccatttccg<br>accaccagcgaaacgccgcgggttgtttataattcgagaacggataagccgtggccggtggcgctttatttgacgc<br>ctgtgagcagtgcgggcggggtggctattaaagctggttcattaattgccgtgcttattttgcgacagaccaacaact<br>ataacagcgatgattttcagtttgtgtggaatatttacgccaataatgatgtggtggtgcccactggcggttgtgatgtt<br>tctgctcgtgatgtcaccgttactctgccggactaccctggttcagtgccaattcctcttaccgtttattgtgcgaaaag<br>ccaaaacctggggtattacctctccggcacaaccgcagatgcgggcaactcgattttcaccaataccgcgtcgtttt<br>caccagcgcagggcgtcggcgtacagttgacgcgcaacggtacgattattccagcgaataacacggtatcgttag<br>gagcagtaggaacttcggcggtaagtctgggattaacggcaaattacgcacgtaccggagggcaggtgactgca<br>gggaatgtgcaatcgattattggcgtgacttttgtttatcaataa
